# A Microfluidic System for Detecting Tumor Cells Based on Biomarker Hexaminolevulinate (HAL): Applications in Pleural Effusion

**DOI:** 10.3390/mi14040771

**Published:** 2023-03-30

**Authors:** Yiran Luan, Lei Li, Xiaoyi Xun, Yang Wang, Xinyue Wei, Yuqun Zheng, Zhijuan Fan, Xuguo Sun

**Affiliations:** 1 School of Medical Laboratory, Tianjin Medical University, Tianjin 300203, China; 2 Department of Laboratory, Tianjin Third Central Hospital, Tianjin 300170, China

**Keywords:** microfluidic chip, tumor cells, pleural effusion, hexaminolevulinate

## Abstract

Malignant pleural effusion is a common clinical problem, which often occurs in cases of malignant tumors, especially in lung cancer. In this paper, a pleural effusion detection system based on a microfluidic chip, combined with specific tumor biomarker, hexaminolevulinate (HAL), used to concentrate and identify tumor cells in pleural effusion was reported. The lung adenocarcinoma cell line A549 and mesothelial cell line Met-5A were cultured as the tumor cells and non-tumor cells, respectively. The optimum enrichment effect was achieved in the microfluidic chip when the flow rates of cell suspension and phosphate-buffered saline achieved 2 mL/h and 4 mL/h, respectively. At the optimal flow rate, the proportion of A549 increased from 28.04% to 70.01% due to the concentration effect of the chip, indicating that tumor cells could be enriched by a factor of 2.5 times. In addition, HAL staining results revealed that HAL can be used to identify tumor cells and non-tumor cells in chip and clinical samples. Additionally, the tumor cells obtained from the patients diagnosed with lung cancer were confirmed to be captured in the microfluidic chip, proving the validity of the microfluidic detection system. This study preliminarily demonstrates the microfluidic system is a promising method with which to assist clinical detection in pleural effusion.

## 1. Introduction

Malignant pleural effusion refers to the excessive pleural effusion caused by the malignant tumor originating in the pleural tissue or the distant metastasis of tumor cells from other body parts to pleural, usually indicating the occurrence and progression of the disease [1]. According to previous work, malignant pleural effusion is mostly caused by metastatic cancer [2]. Lung cancer remains the most common cause of malignant pleural effusion caused by metastatic cancer: about 50% of patients present with excessive pleural effusion during the course of lung cancer [3,4]. Exfoliated tumor cells found in pleural effusion can be seen as a sign of advanced cancer and are usually associated with poor prognosis of patients with lung cancer [5,6]. Therefore, effective isolation and diagnosis of malignant cells in a large volume of pleural effusion are crucial to progression and prognosis.

At present, cytological examination is the most common diagnostic method in clinical pleural effusion detection, with high specificity but low sensitivity. The diagnostic rate for traditional pleural cytology based on morphology has been intensively reported, with a mean sensitivity of about 60%, indicating the rate of missed diagnosis can reach 40% [7]. Based on the challenges that exist in conventional clinical work, lots of researchers are devoted to improving the efficiency of diagnosis. It is reported that immunocytochemistry can promote the accuracy of tumor cells [8]. However, it is usually based on a broad range of markers, and thus is time consuming. Despite the obvious advantage of high throughput, it is rare for flow cytometry to diagnose malignancy in pleural effusion. The size and cohesiveness of malignant cells are believed to limit its wider use. Nowadays, due to the spurt of progress in computer technology, a computer assistant diagnosis system based on intelligent algorithm recognition for identifying pleural effusion cells has also been exploited [9]. However, the results showed that the specificity of the trained system can reach 99.40%, while the sensitivity was only 87.97%. Meanwhile, the data set used for training involved digitized cytology images captured from the samples on the glass slide with classical Papanicolaou staining. Due to the subjective characteristics of Papanicolaou staining, the test results contained a certain rate of missed diagnosis.

Microfluidic chip technology has been widely applied in the detection of tumor cells in different body fluids, such as blood [10,11,12], urine [13,14,15], sputum [16], ascites [17,18] and pleural effusion. Che et al. described a centrifuge chip with microchambers to employ microscale vortices for the isolation and concentration of cancer cells and mesothelial cells [19]. This chip can increase the purity greater than 65-fold in malignant pleural effusion samples. Nonetheless, normal mesothelial cells were included in purity measurements, as they share a similar size with tumor cells. In a more recent study, Zhao et al. designed a microfluidic chip with straight channels of different widths for the size-based isolation of tumor cells and clusters [20]. The tumor cell clusters were successfully collected from the outlet with a recovery rate of 80%. However, this study did not give a detailed description of the performance of microfluidic chip on separating tumor cells from pleural effusion. Shi et al. proposed a microfluidic chip with contraction−expansion array structure [21]. Through introducing a viscoelastic fluid, the device can achieve size-selective separation of target tumor cells from pleural effusion. The contraction–expansion array can be used as an effective tool to expand the distance between the tumor cells and initial samples. As the results showed, a separation efficiency of more than 90% was achieved for the simulated samples in this study. However, clinical samples are required for further validation.

Spiral inertial microfluidics is a passive and label-free enrichment tumor cell technology based on the principle of fluid dynamics. Tsou et al. introduced a spiral microfluidic device, which can enable 90% of lung cancer cells to flow to the target outlet and increase the purity of cancer cells identified in pleural effusion by 6 to 24 times [22]. Zhu et al. proposed a microfluidic chip with a slanted spiral channel [23]. As a result, more than 85% of the lung cancer cells in simulated pleural effusion can be recovered. Nevertheless, the spiral chip realizes the enrichment of tumor cells by means of cell size. Normal mesothelial cells with large size in pleural effusion often interfere with the isolation performance, affect the purity of the target cells and pose challenges for subsequent analysis.

At present, the chip used for malignant pleural effusion analysis mainly focuses on the isolation and purification of tumor cells. Although the purity of chip-concentrated tumor cells has been greatly increased, specific identification of tumor cells is still required after concentration. 5-aminolevulinic acid (5-ALA), as a natural amino acid, has been demonstrated to biosynthesize an intermediate fluorescent production protoporphyrin-IX (PpIX) via heme synthesis pathway in vivo [24]. Generally, there are few accumulations of intracellular PpIX. Therefore, it is difficult to detect the fluorescence of PpIX. When exogenous 5-ALA or its derivatives are provided, the endogenous PpIX accumulates in the mitochondria. This is due to the different expression of the key enzyme in the heme pathway between the tumor cells and normal cells, leading to the higher PpIX accumulation in tumor cells [25]. In this study, the 5-ALA derivative hexaminolevulinate (HAL) is used to identify tumor cells in pleural effusion.

The purpose of this study is to design a label-free microfluidic chip based on the hydrodynamic principle to separate tumor cells from red blood cells (RBCs) in pleural effusion and employ HAL staining for the further identification, thus building a microfluidic detection system for tumor cells in pleural effusion.

## 2. Materials and Methods

### 2.1. Cell Culture

Human lung cancer cell line A549 and human mesothelial cell line Met-5A (Cell Resource Center, Shanghai Institutes for Biological Sciences, Chinese Academy of Sciences) were cultured as tumor cells and normal cells in pleural effusion, respectively. A549 cells and Met-5A were cultured in DMEM medium and RPMI-1640 medium (Thermo Fisher Scientific Inc., Waltham, MA, USA) containing 10% fetal bovine serum and 1% penicillin/streptomycin (Thermo Fisher Scientific Inc., Waltham, MA, USA), as previously described [26,27]. The cells were cultured at 37 °C under an atmosphere with 5% CO_2_.

### 2.2. Wright–Giemsa Staining

A small drop of cell suspension was smeared on a microscope slide. Next, 0.5 mL Wright–Giemsa dye solution (Baso Diagnostics Inc., Zhuhai, China) was added to the slide for 1 min. Then, 1 mL Phosphate Buffer Solution (PBS) (Golden Clone (Beijing) Biotechnology Co., Ltd., Beijing, China) was added for an additional 5 min. Cell morphology was observed via optical microscopy.

### 2.3. HAL-Induced Fluorescence

#### 2.3.1. HAL-Induced Fluorescence in Cultured Cells

When the cells reached 80% confluence, they were detached from the culture flask. Then, DMEM medium without fetal bovine serum was used to suspend cells. Cell suspension was mixed with 400 μM HAL solution at the ratio of 1:1 (*V*/*V*) and cultured in a standard incubator at 37 °C in the dark [28]. Met-5A was cultured as the negative control and stained with the same steps described above. Fluorescence microscopes (ECLIPSE Ni-U, ECLIPSE Ti-U, Nikon, Tokyo, Japan) were used to observe the HAL-induced fluorescence production in cells.

#### 2.3.2. HAL-Induced Fluorescence in Clinical Samples

Once the clinical samples have been obtained, it is recommended to conduct HAL staining immediately. After fully shaking and mixing the pleural effusion sample, 100 μL clinical sample was mixed with 400 μM HAL solution at the ratio of 1:1 (*V*/*V*). The mixture was incubated at 37 °C in the dark. Fluorescence in clinical samples was observed using a fluorescence microscope (ECLIPSE Ni-U, Nikon, Tokyo, Japan).

### 2.4. Sorting Principle of Microfluidic Chip

The microfluidic chip used in this work was designed by AutoCAD software. Suzhou Institute of Nano-Tech and Nano-Bionics, Chinese Academy of Sciences was trusted to manufacture the chip. The separation function of the chip is based on the principle of deterministic lateral displacement. The micropillar in deterministic lateral displacement array is arranged at an angle of 15° inside the chip. Particles with different sizes flow through the array in different ways. Cells which are smaller than the critical diameter flow in the original stream generally do not undergo lateral displacement. Thus, they are collected from the lateral outlet, also called the small-size outlet. However, the cells larger than the critical diameter are laterally displaced, deflecting to the other direction and flowing through the capture array. The capture array contains capture sites which are designed according to the size of tumor cells in order to capture tumor cells within a certain size range and finally ensure the preliminary separation of tumor cells in pleural samples.

### 2.5. Clinical Samples

The pleural effusion samples were collected from patients diagnosed with lung cancer according to the criteria of the clinical diagnosis and treatment guidelines of lung cancer. The study was conducted in accordance with the Declaration of Helsinki, and the protocol was approved by the Ethics Committee of Tianjin Chest Hospital (project identification code: 2023LW-003). Next, 4% paraformaldehyde (Labgic Technology Co., Ltd., Beijing, China) was added to fix the cell morphology. Before pumping into the chip, the pleural effusion samples were diluted to 1 × 10^6^ cells/mL with PBS.

## 3. Results

### 3.1. Generation of HAL-Induced Fluorescence in Tumor Cells

**Figure 1:**
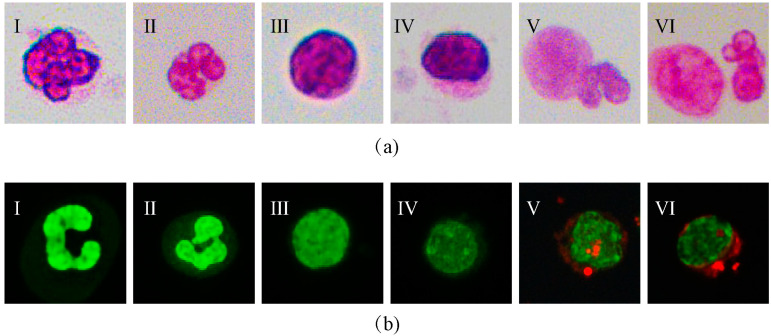
(**a**) Microscopic images of different cells stained with Wright–Giemsa. I–II: granulocyte, III–IV: Met-5A, V–VI: A549. (**b**) Microscopic images of different cells stained with hexaminolevulinate (HAL). I–II: granulocyte, III–IV: Met-5A, V–VI: A549.

Human lung adenocarcinoma cell line A549 was selected as the tumor cells of the pleural effusion, while the human mesothelial cell line Met-5A and neutrophils were selected as the normal cells for comparison against tumor cells. Wright–Giemsa and HAL staining were performed to determine the cell types. Using Wright–Giemsa staining, we confirmed that the total area of these three types of cells was close to each other. In terms of nuclear morphology, the morphology of neutrophils was obviously different from the other two kinds of cells. However, it is arduous to determine the biological characteristics of A549 and Met-5A in terms of nuclear sizes and curvatures around the nucleus. In addition, the intracellular biomarker HAL was labeled to determine the cell types. The results revealed that red fluorescence was visible in A549 cells, while negative fluorescence reaction was found in Met-5A and neutrophils (Figure 1). Taken together, the results demonstrated that HAL has the ability to identify tumor cells from non-tumor cells.

### 3.2. Construction of the Microfluidic System

**Figure 2:**
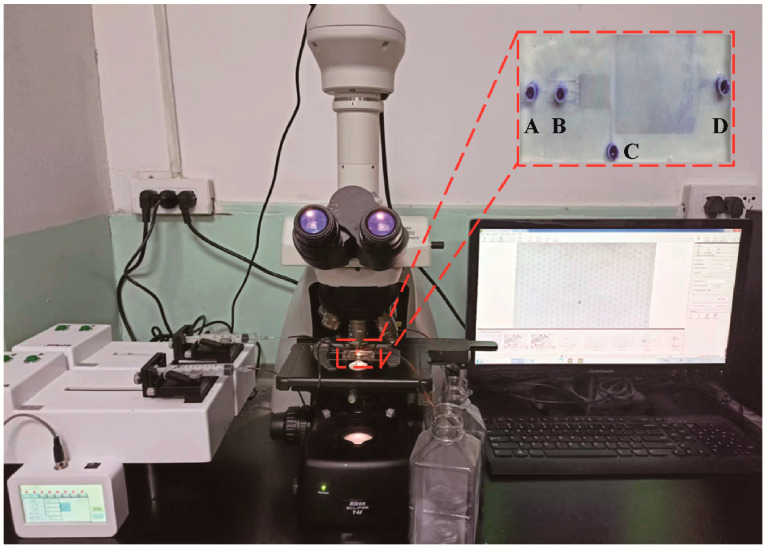
A prototype of microfluidic detection system for tumor cells in pleural effusion. The inset shows the microfluidic chip. A: cell suspension inlet, B: PBS solution inlet, C: small-size cell outlet, D: large-size cell outlet.

The microfluidic system constructed for the detection of tumor cells in pleural effusion is made up of a control system, a drive system, a microfluidic chip, an optical microscope and a software system. The control system includes a syringe pump controller, which controls the syringe pumps. Two syringe pumps are used to inject PBS solution and cell suspension into the microfluidic chip. An optical microscope with a charge-coupled device is used to capture the cell morphology in the microfluidic chip. The cell characteristic is displayed by the image analysis software (Figure 2).

The microfluidic chip consists of two inlets (A, B) and two outlets (C, D). The inlet A is the cell suspension inlet; the inlet B is the PBS inlet. The outlet C is designed as the small-size cell outlet, from which we intend to collect the RBCs. Target tumor cells flow through the chip and are captured in the chip. After identification, the cells flow out through the outlet D. The chip includes an isolation module and a capture module. There are 72 micropillars in isolation module and 11,454 capture sites in the capture module. The specific structure of the deterministic lateral displacement array in isolation module and capture array in capture module is clearly shown in Figure S1.

### 3.3. Optimization of the Flow Rates

**Figure 3:**
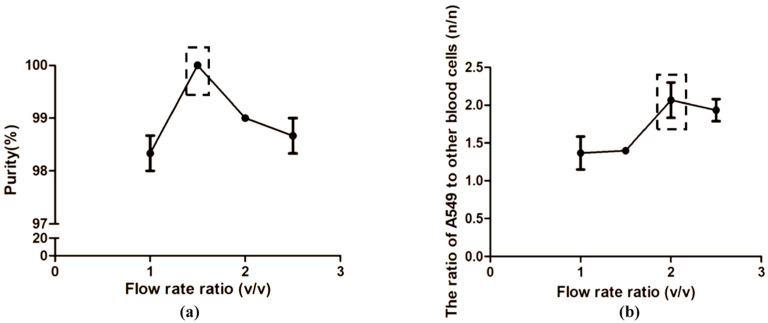
Optimization of the flow rates. (**a**) Isolation purity of Red blood cells (RBCs) from the outlet C at different flow rate ratios; (**b**) the ratio of A549 to other blood cells at different flow rate ratios in microfluidic chip. The parts enclosed by dotted lines are the best isolation performance of the chip with the optimal flow rate ratio.

In this study, A549 was employed to mimic the malignant exfoliated tumor cells in clinical pleural effusion. To mimic clinical pleural effusion, tumor cells were spiked in normal peripheral blood with a certain dilution and pumped into the chip together at a certain flow rate. Based on the previous work of our group (Figure S2), we set the concentration of cells in suspension around 1 × 10^6^ cells/mL and the proportion of A5459 in suspension around 30%. The flow rate of the simulated clinical sample was fixed at 2 mL/h. The PBS buffer was injected into the chip at a flow rate from 2 mL/h to 5 mL/h. To determine the optimal flow rate, we calculated the proportions of the tumor cells and RBCs captured in the capture area, as well as the proportions of the tumor cells and RBCs collected from the outlet C under the different velocities. The results showed that, with different PBS buffer flow rates, the percentage of RBCs in cell suspension collected from outlet C ranged from 98% to 100%. Only a few tumor cells were observed in cell suspension collected from outlet C (Figure S3). Inside the chip, the ratio of tumor cells to RBCs in the capture area was the highest when the flow rate of buffer was 4 mL/h. We selected the flow rate of PBS buffer at 4 mL/h as the optimal rate for subsequent analysis. At this time, the ratio of injected cell suspension and PBS was 1:2 (Figure 3).

### 3.4. HAL Fluorescence Detection

Traditional HAL fluorescence reaction and HAL fluorescence reaction based on microfluidic chip were evaluated in lung cancer cell line A549 and mesothelial cell line Met-5A, respectively. In our previous work, we set a series of concentration gradients (50 µM, 100 µM, 150 µM, 200 µM, 250 µM, 300 µM) and incubation time gradients (0.5 h, 1 h, 2 h, 4 h, 6 h) to determine the optimal incubation condition. As can be seen from Figures S4 and S5, when the HAL concentration was 200 µM and the incubation time was 2 h, intensive red fluorescence was observed in A549 cells. For that reason, 200 µM concentration and 2 h dark incubation of HAL were chosen for comparison between different cell lines. Traditional HAL fluorescence reaction was performed on microscope slides. The results clearly showed the differential expression of HAL-induced red fluorescence between normal mesothelial cell lines Met-5A and tumor cells A549.

**Figure 4:**
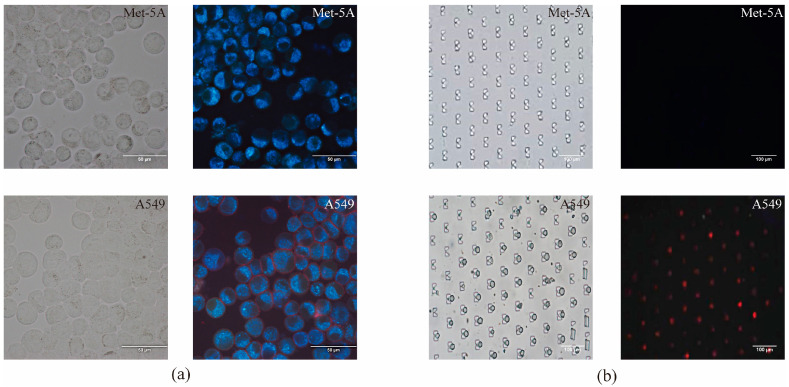
Microscopic images of Met-5A and A549 stained with HAL in (**a**) Slides and (**b**) microfluidic chip. A549 are observed with red fluorescence, which is distinguished from the unlabeled Met-5A. The images of slides are captured at 40× objective and the images of microfluidic chip are captured at 20× objective.

We also evaluated the HAL staining performance in a microfluidic chip. The total flow rate was set to 6 mL/h (*v_A_*:*v_B_* = 1:2) based on the Section 3.3. As shown in Figure 4b, we observed the red fluorescence in the cytoplasm of A5459 cells captured in the chip. However, positive fluorescence was not found in the cytoplasm of Met-5A captured in the chip at the same incubation time and concentration of HAL. The results indicated the feasibility of HAL performed using a microfluidic chip.

### 3.5. Concentration Performance of the Microfluidic System

To evaluate the concentration capability of the constructed microfluidic system, granulocytes, A549 and RBCs were fully mixed to simulate clinical pleural effusion until the A549:RBC was at the ratio of 1:2.6. After mixing them up, the cell suspension was injected into the chip at 2 mL/h, while the PBS was injected into the chip at 4 mL/h. The number ratio of A549:RBC was 2.3:1 in the capture area of the microfluidic chip. The proportion of A549 increased from 28.04% to 70.01%. The results showed that the tumor cells were concentrated by a factor of 2.5 times (Table 1).

### 3.6. Validation of Clinical Sample

**Figure 5:**
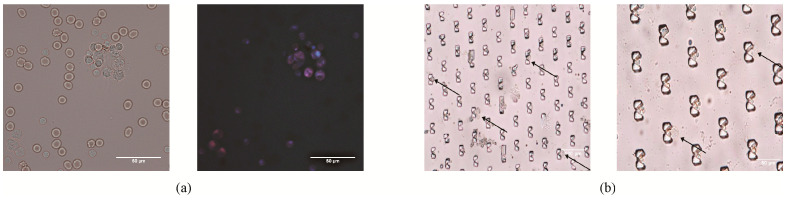
(**a**) Clinical pleural effusion sample stained with HAL. The images are captured at 40× objective. (**b**) Images of tumor cells in pleural effusion of lung cancer patients captured in microfluidic chip. The images are captured at 20× objective and 40× objective, respectively. The arrows show the suspicious tumor cells captured in the chip.

We performed HAL staining in malignant pleural effusion samples to verify the practical application in clinical detection. As shown in Figure 5a, red fluorescence was found in malignant tumor cells, while negative fluorescence reaction was found in RBCs and leukocytes under the optimal concentration and incubation time of HAL (Figure 5a).

To further verify the capture capability of clinical samples of the microfluidic chip, we diluted the malignant pleural effusion obtained from the lung cancer patients to 1 × 10^6^ cells/mL with PBS. The total flow rate was set to 6 mL/h (*v_A_*:*v_B_* = 1:2) to complete the experiment. We preliminarily identified the cells according to the morphology. The results showed that RBCs, pathogenic microorganisms and other visible components were excluded through the outlet C, while suspicious large-size cells were captured in the trap array inside the microfluidic chip (Figure 5b).

## 4. Discussion

In recent years, more and more studies have turned their attention to the application of microfluidic technology in clinical pleural effusion detection. The extensively implemented microfluidic chips are based on the hydrodynamics principle for the size-based isolation and concentration of rare tumor cells from large volume pleural effusion samples. However, normal mesothelial cells and malignant tumor cells are similar in size, which may affect the purity of tumor cells. Besides, the tumor cells in the clinical samples purified by microfluidic chips still require specific identification to determine the final diagnosis. To resolve this problem, a capture area was designed as the complement to the label-free isolation in this study. The tumor biomarker HAL was employed to identify the cells captured in the capture area.

Firstly, we discussed the effect of the rate of cell suspension and PBS solution injected into the microfluidic chip on the performance of concentrating tumor cells. On the premise of keeping the flow rate of cell suspension at 2 mL/h, we changed the flow rate of PBS buffer and calculated the proportions of tumor cells that flew through the outlet C and the proportions of tumor cells captured in the microfluidic chip. The results showed that when the PBS flow rate changed within a certain range, the purity of the RBCs collected from the small-size cell outlet could be maintained at a high level (98–100%) constantly, indicating that changing the rate ratio of the solution would not easily miss the tumor cells in cell suspension. At the same time, the ratio of tumor cells to blood cells captured in the chip with the increase in the total volume of the solution injected into microfluidic chip was analyzed. The results showed that when the PBS flow rate was 4 mL/h, the ratio of tumor cells to blood cells in the capture area was the largest. We considered that the total flow rate is 6 mL/h, while the cell suspension is 2 mL/h, and the PBS buffer is 4 mL/h in the appropriate conditions. The rates were adopted for subsequent analysis.

In addition to rare tumor cells, we can observe a variety of cells including granulocytes, lymphocytes, mesothelial cells, and erythrocytes in pleural effusion [29]. The number of erythrocytes varies depending on the severity of the disease and the degree of vascular damage at the time of sampling. Wright–Giemsa staining, which is commonly implemented on clinical work, highly relies on the opinions of pathological experts when identifying pleural effusion cells. Among several cell types in pleural effusion, granulocytes, tumor cells and mesothelial cells have a similar size. Since lung cancer is the most common cause of malignant pleural effusion [30], human lung cancer cell line A549 cultured in vitro was selected as the tumor cell in pleural effusion. Granulocytes, A549 and Met-5A were dyed with Wright–Giemsa. Observing the morphological characteristics of cells using a microscope, we found that the nuclear morphology of granulocytes is significantly different from A549 and Met-5A. However, A549 has similar characterization to nuclear morphology with Met-5A. The results suggested that the presence of mesothelial cells in pleural effusion may interfere with the observer and cause certain errors.

The chip based on the deterministic lateral displacement work principle is able to reduce the number of small-size interfering cells in pleural effusion samples, effectively avoid the interference of chaotic background cells on the detection of target cells and as enrich tumor cells in pleural effusion. Investigating the proportions of target tumor cells captured in the chip to other cells, we found that the chip has the ability to concentrate pleural effusion samples by 2.5 times, greatly reducing the burden of subsequent analysis.

5-ALA utilizes the differences in the heme synthetic pathway to distinguish tumor cells from normal cells. It has been reported that 5-ALA was applied in vitro to detect the PpIX-induced fluorescence in human mesothelioma cell lines and human lung cancer cell lines, and clear red fluorescence was observed in human lung cancer cell line A549 [31]. HAL, a hexyl ester derivative of 5-ALA, was used in this study. Because of its lipophilicity, HAL has the advantage of inducing the accumulation of PpIX in vitro with lower concentration and shorter incubation time compared with 5-ALA [32,33,34,35]. In this work, human mesothelial cell line Met-5A and human lung cancer cell line A549 were incubated with HAL. The results demonstrated that we can distinguish mesothelial cells from tumor cells based on the specific red fluorescence induced by HAL.

To further verify the performance of 5-ALA on the detection of tumor cells in microfluidic chip, we mixed A549 cells with peripheral blood cells and fixed the proportion of A549 in total cells around 30% to simulate the proportion of tumor cells in clinical malignant pleural effusion samples. Then, the cell suspension was injected into the microfluidic chip using the syringe pump. The results showed that A549 captured in the microfluidic chip still expressed stable red fluorescence, while Met-5A, cultured as normal cells in pleural effusion, had a negative fluorescence reaction. From these results, it is clear that specific fluorescence produced by tumor cells can be observed in pleural effusion samples in our chip. Our results suggested that this method can provide a diagnosis method for clinical application to distinguish intrapleural mesothelial cells and tumor cells. In order to confirm the clinical applicability, we collected pleural effusion samples of lung cancer patients and performed HAL staining in clinical samples. We observed red fluorescence in malignant tumor cells in pleural effusion, which could be easily distinguished from RBCs and leukocytes with a negative fluorescence reaction. We also used the microfluidic chip to analyze cell types in samples. The results verified that tumor cells were visible in the capture area of the microfluidic chip. Collectively, the results supported the practicability of HAL in clinical pleural effusion detection and proved that the microfluidic chip designed in this work has the potential to capture tumor cells in pleural effusion of lung cancer patients.

When comparing our results to previous studies, it must be pointed out that the microfluidic detection system we built for pleural effusion detection is able to identify tumor cells on the basis of preliminary separation. The universal tumor biomarker HAL applied in this study is able to distinguish cells of similar size based on the metabolic characteristics of malignant tumor cells. It is notable that HAL staining omits the complex staining steps. Besides, HAL is different from the cell surface and intracellular markers exclusively expressed on malignant cells. Sometimes, the decreased expression of the markers may lead to missed diagnosis. The main limitation of our work is the lack of the application of HAL in clinical samples processed by microfluidic system; we intend to combine it with image recognition algorithms in our future research. One important future direction of our study is to build an image-based analysis system to realize automatic detection of tumor cells in pleural effusion by identifying the features of cell fluorescence images. In conclusion, the microfluidic system established in this work provides a new detection method for tumor cells in pleural effusion.

## 5. Conclusions

We set up a label-free microfluidic chip based on cell sizes for sorting and enriching tumor cells in pleural effusion, which can aggregate small-size RBCs and allow them to flow out through lateral microchannel, while the large-size cells, such as lung adenocarcinoma cells A549, mesothelial cells Met-5A and neutrophils, flow through the capture area inside the microfluidic chip. Our chip has the ability to concentrate tumor cells by a factor of 2.5 times and separate RBCs from other cells in pleural effusion. Meanwhile, we add a capture area and employ HAL for the specific identification. We preliminarily prove the availability of HAL in clinical pleural effusion detection. The whole detection system is a one-step system without any additional complicated processing steps. It is suitable for isolation and detection of tumor cells in pleural effusion.

To the best of our knowledge, this is the first time that the microfluidic chip combined with tumor biomarker HAL has been used to isolate and identify the tumor cells in malignant pleural effusion. It paves a new way to detect tumor cells in pleural effusion.

## Figures and Tables

**Table 1:** Performance of the concentration capability of microfluidic chip.

	Before Concentration		After Concentration	
	A549 (%)	RBC (%)	A549 (%)	RBC (%)
Proportion	28.04 ± 3.96	71.96 ± 3.96	70.01 ± 9.36	29.99 ± 9.36

## Data Availability

The data presented in this study are available on request from the authors.

## Supplementary Materials

The following supporting information can be downloaded at: https://www.mdpi.com/article/10.3390/mi14040771/s1, Figure S1: Design of the microfluidic chip. Figure S2: Microscopy images of cells sorted from outlet C at different orders of magnitude; Figure S3: Microscopy images of cells sorted from outlet C; Figure S4: Microscopy images of A549 stained with HAL at different incubation time; Figure S5: Microscopy images of A549 stained with HAL at different concentrations.
