# Supplementary material for: A Microfluidic System for Detecting Tumor Cells Based on Biomarker Hexaminolevulinate (HAL): Applications in Pleural Effusion
Source: Micromachines (Basel). 2023 Mar 30;14(4):771. doi: 10.3390/mi14040771 (PMC10146342; doi:10.3390/mi14040771)
Supplement: Supplementary file 1 [file micromachines-14-00771-s001.zip › micromachines-2201100-supplementary.pdf]

Supplementary Materials: A Microfluidic System for Detecting Tumor Cells Based on Biomarker Hexaminolevulinate (HAL): Applications in Pleural Effusion

Yiran Luan, Lei Li, Xiaoyi Xun, Yang Wang, Xinyue Wei, Yuqun Zheng, Zhijuan Fan and Xuguo Sun

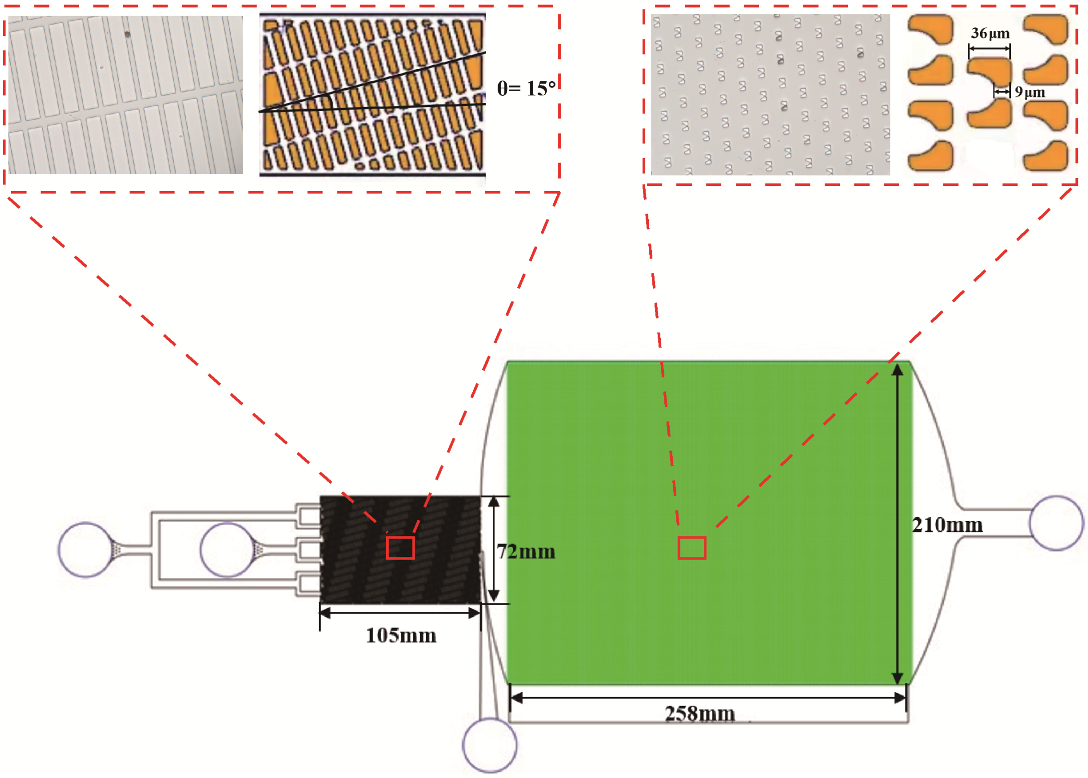

Figure S1. Design of the microfluidic chip. The insets show the detailed structure and main parameters of the isolation module and capture module.

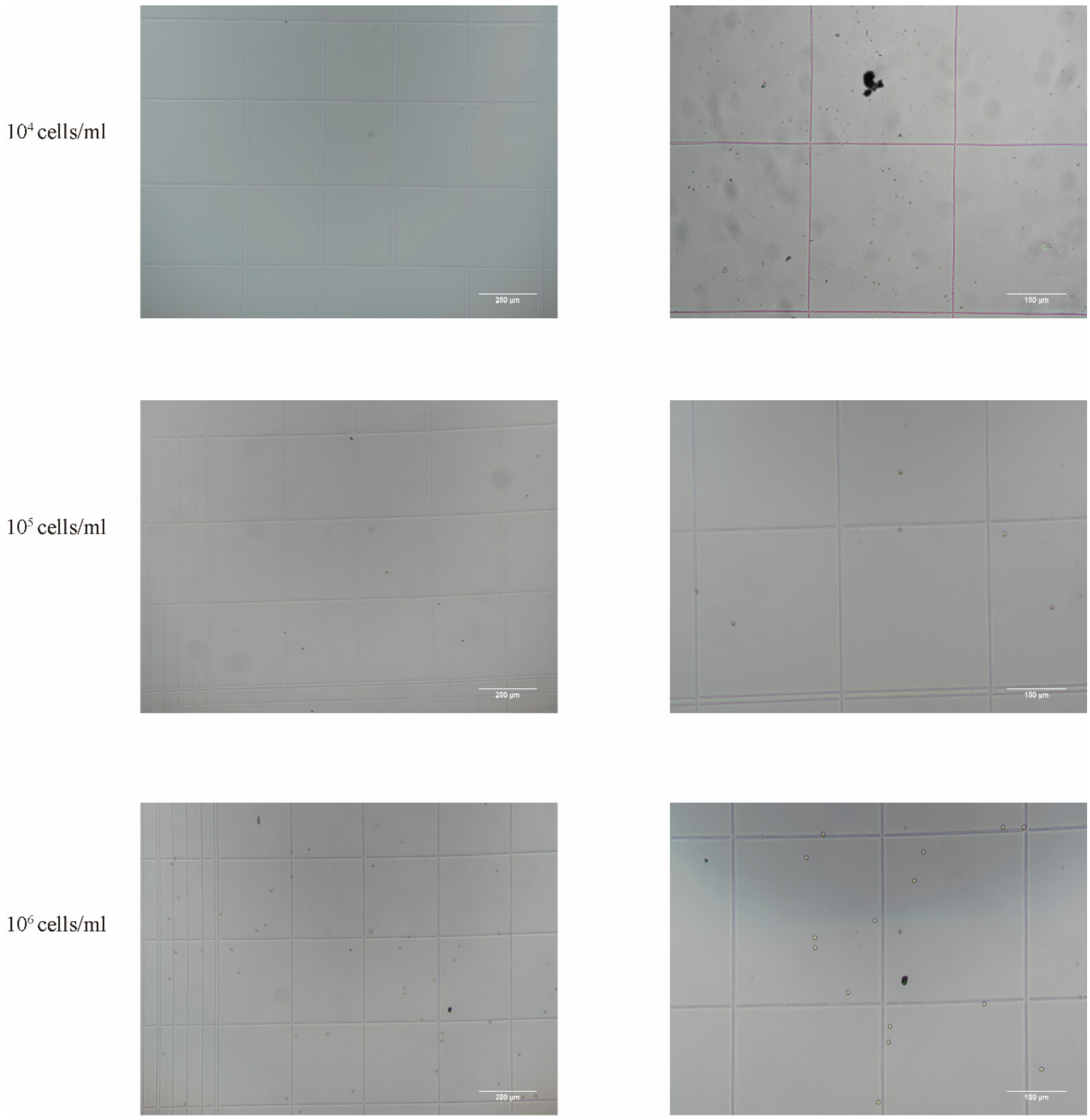

Figure S2. Microscopy images of cells sorted from outlet C at different orders of magnitude. The images are captured at 10× objective and 20× objective, respectively.

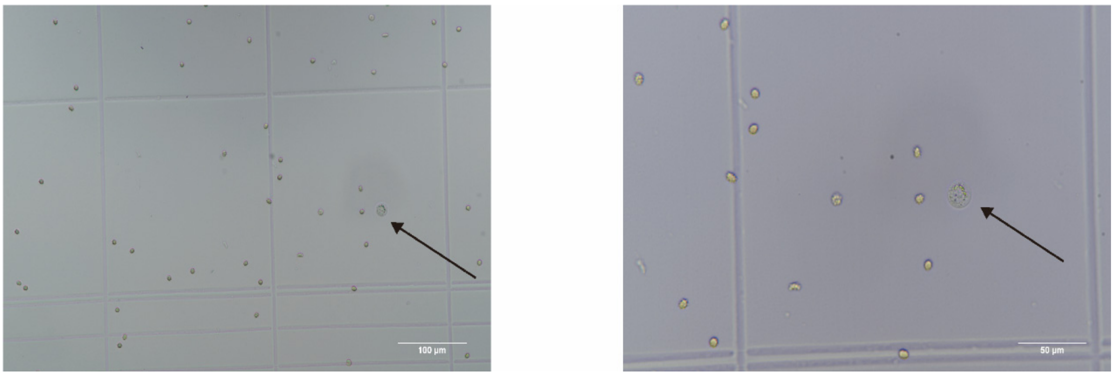

Figure S3. Microscopy images of cells sorted from outlet C. The images are captured at 20× objective and 40× objective, respectively. The arrows show the tumor cells A549.

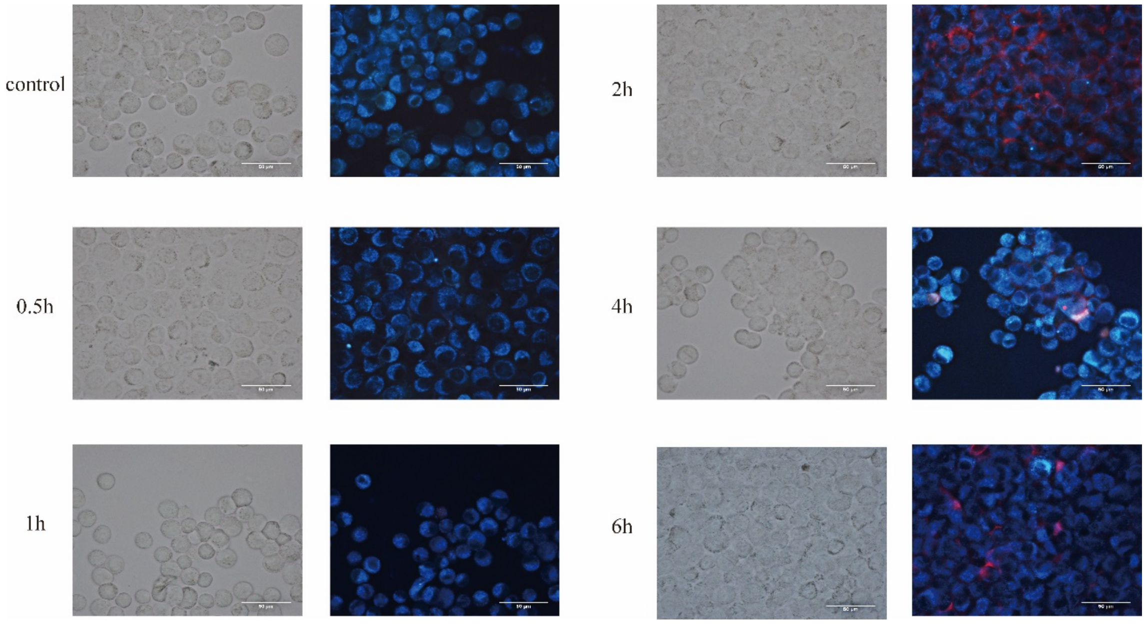

Figure S4. Microscopy images of A549 stained with HAL at different incubation time. The concentration of HAL is 200μM. The images are captured at 40× objective.

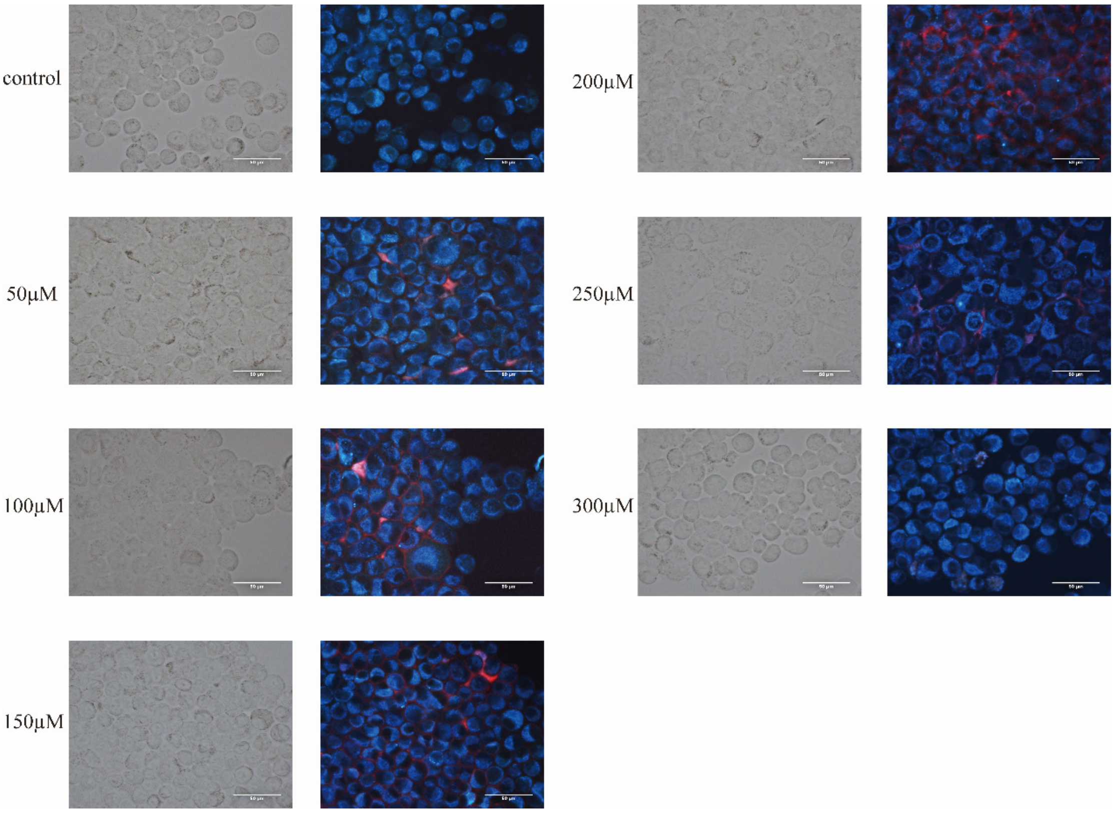

Figure S5. Microscopy images of A549 stained with HAL at different concentrations. The incubation time is 2 hours. The images are captured at 40× objective.
