# Supplementary figures and images for: A Microfluidic System for Detecting Tumor Cells Based on Biomarker Hexaminolevulinate (HAL): Applications in Pleural Effusion
Source: Micromachines (Basel). 2023 Mar 30;14(4):771. doi: 10.3390/mi14040771 (PMC10146342; doi:10.3390/mi14040771)

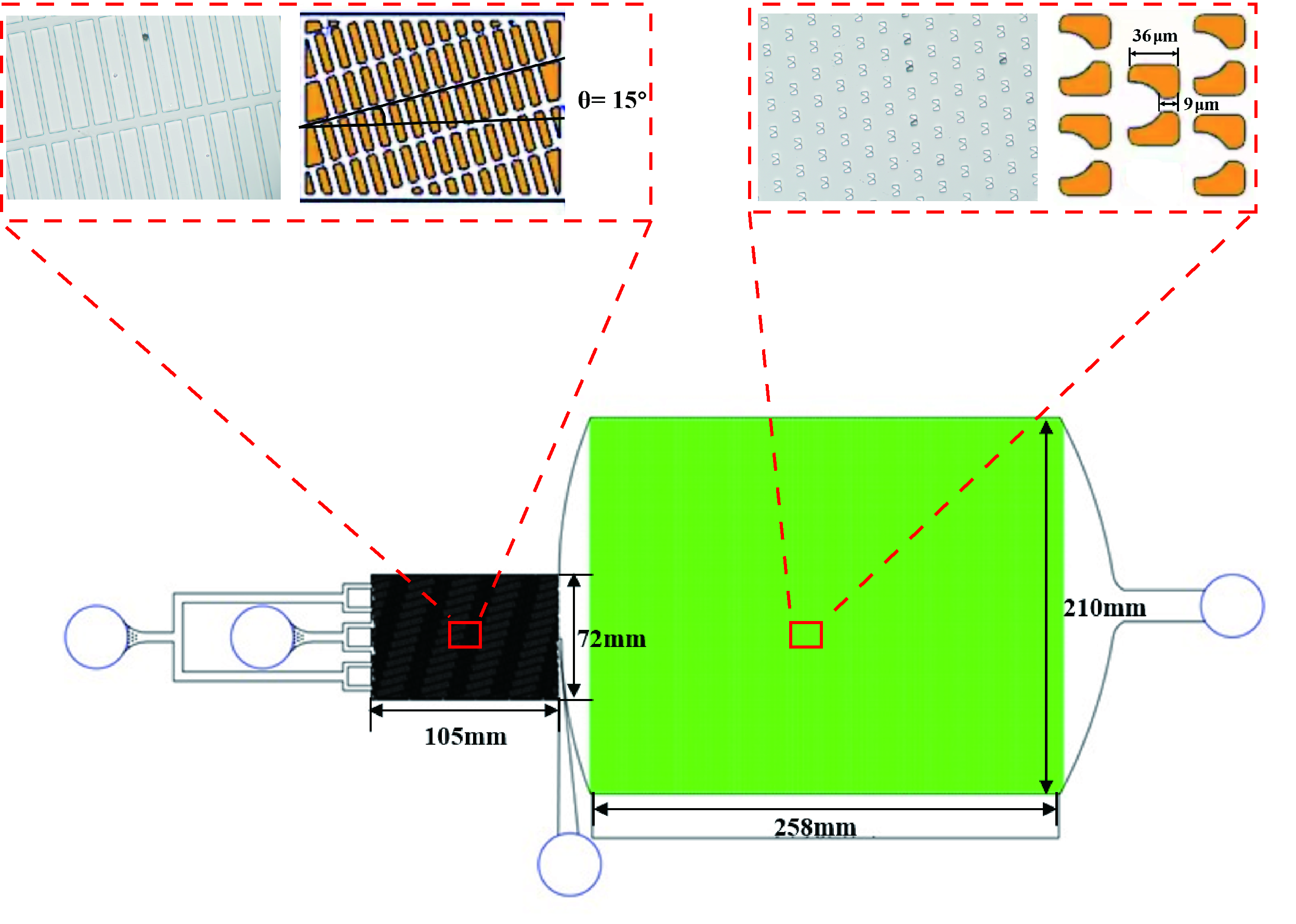

Supplement: Supplementary file 1 [file micromachines-14-00771-s001.zip › Figure S1.tif]

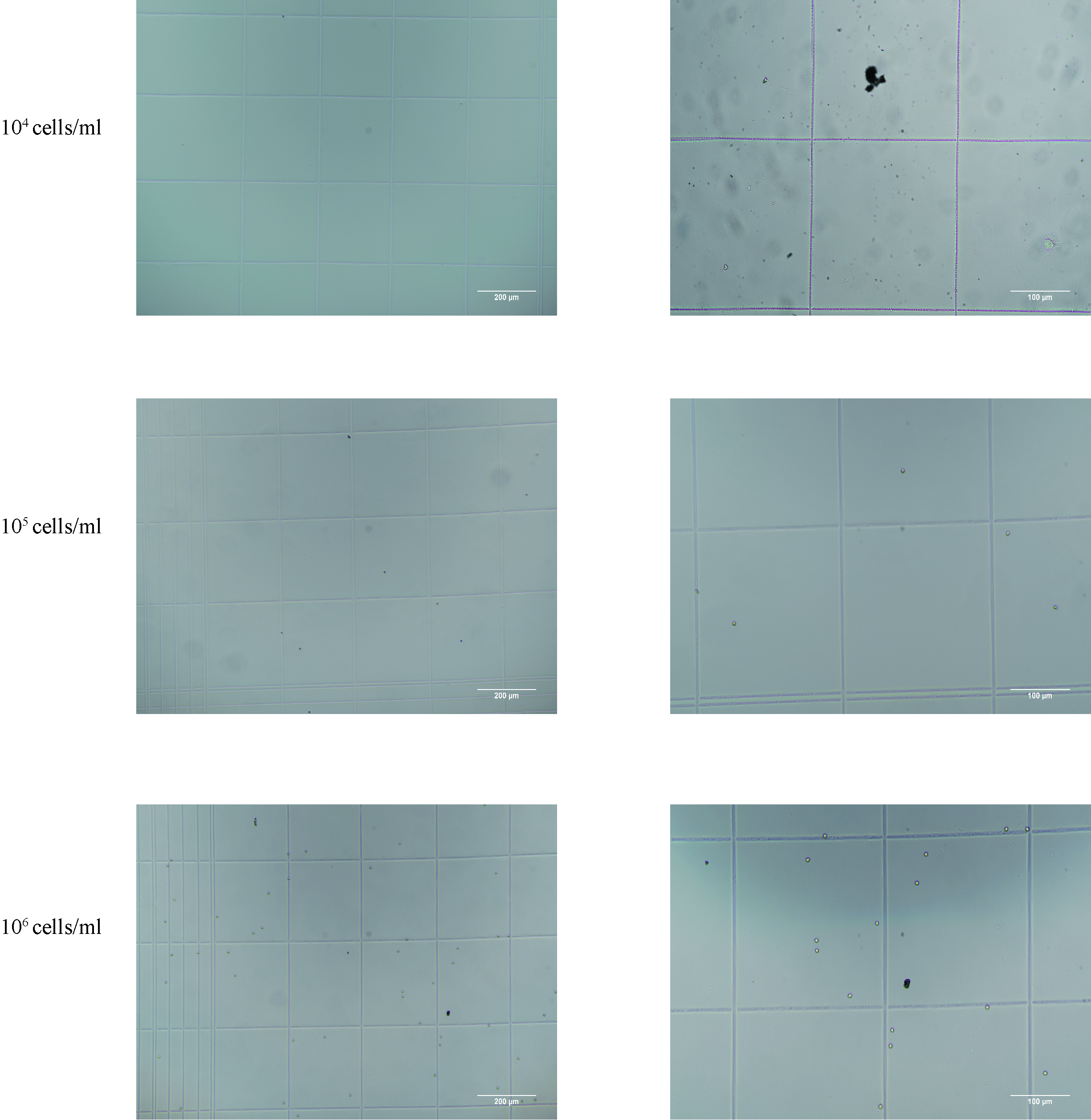

Supplement: Supplementary file 1 [file micromachines-14-00771-s001.zip › Figure S2.tif]

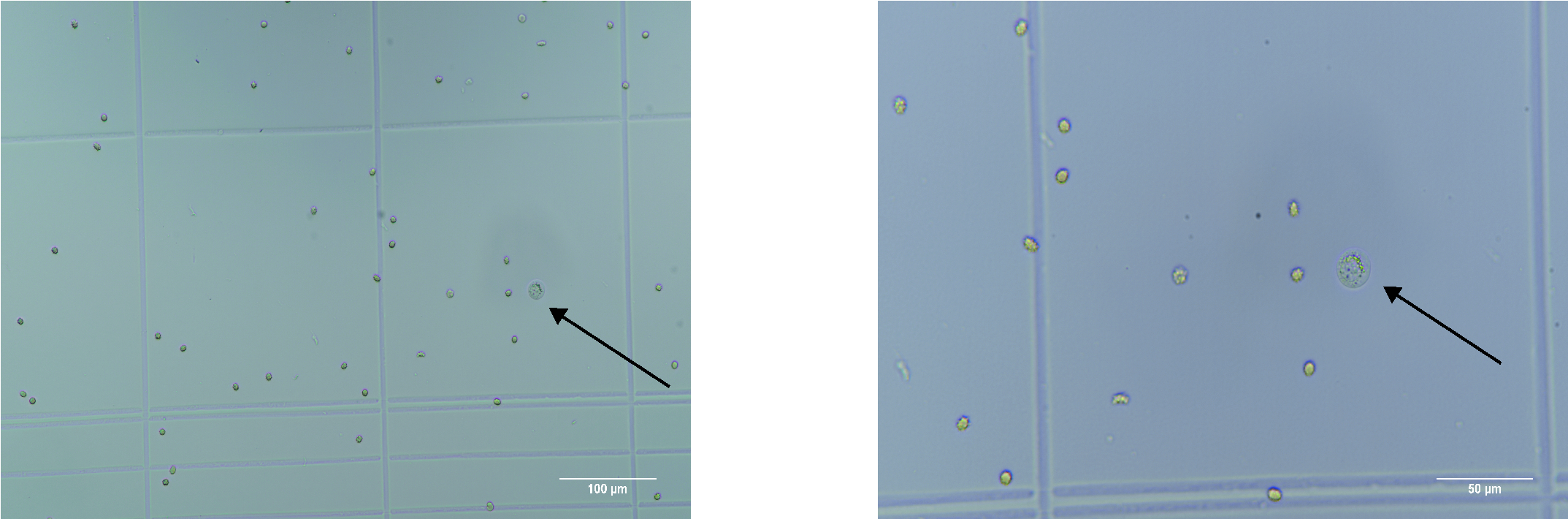

Supplement: Supplementary file 1 [file micromachines-14-00771-s001.zip › Figure S3.tif]
